# Manganese‐enhanced MRI during remotely induced myocardial ischemia reperfusion injury in male mice

**DOI:** 10.14814/phy2.70442

**Published:** 2025-07-03

**Authors:** Matic Pusovnik, Michiel Algoet, Willy Gsell, Stefan Janssens, Wouter Oosterlinck, Uwe Himmelreich

**Affiliations:** ^1^ Biomedical MRI, Department of Imaging and Pathology KU Leuven Leuven Belgium; ^2^ Department of Cardiovascular Sciences KU Leuven Leuven Belgium; ^3^ Bruker Belgium Kontich Belgium

**Keywords:** ischemia–reperfusion injury, left anterior descending artery, manganese enhanced magnetic resonance imaging, myocardial perfusion, myocardial remodeling

## Abstract

Early assessment of myocardial viability post‐ischemia is crucial to mitigate adverse remodeling and optimize therapy. Current noninvasive methods like late gadolinium enhancement (LGE) MRI may overestimate infarct size. Manganese‐enhanced MRI (MEMRI) emerged as a promising alternative, offering greater specificity in assessing myocardial damage. We evaluated MEMRI alongside LGE and histology in a murine ischemia–reperfusion model using a novel “in‐scanner” remote occlusion technique for real‐time imaging during acute ischemia. Male C57BL/6 mice (*n* = 16) underwent left anterior descending artery occlusion (*n* = 7), sham surgery (*n* = 6), or no intervention (*n* = 3). MEMRI (0.1 mmol/kg MnCl_2_) during ischemia (0–60 min) and LGE (0.1 mmol/kg Gd‐DOTA, 24 h post‐surgery) quantified perfusion deficits and infarct size. MEMRI detected acute hypo‐perfusion (lateral wall signal reduction: *p* < 0.01 vs. septal), confined to the occluded territory, while LGE overestimated infarct size (*p* = 0.0225 vs. histology). Ischemic mice showed adverse remodeling with reduced ejection fraction (61.37% vs. 71.92%, *p* < 0.01). MEMRI‐derived perfusion deficits correlated with functional decline and histology‐confirmed infarcts. Pre‐occlusion T_1_ times did not differ between ischemic and sham groups (*p* = 0.85), confirming technique specificity. MEMRI enables early, accurate ischemic injury detection and predicts cardiac dysfunction, outperforming LGE in infarct size determination. Our remote occlusion technique facilitates real‐time perfusion assessments, enhancing preclinical myocardial ischemia studies.

## 1 INTRODUCTION

Ischemic heart disease remains the major cause of mortality and morbidity worldwide. When myocardial blood flow is compromised, a progressive cascade of events causes irreversible myocyte necrosis and loss of contractile force as myocytes are replaced by fibroblasts and nonviable scar tissue. Timely and noninvasive assessment of myocardial viability, and accurate infarct size quantification post‐myocardial ischemic injury are therefore vital for evaluating optimized treatment strategies for cardioprotection.

Cardiovascular magnetic resonance imaging (cMRI) emerged as an indispensable clinical and research tool for quantitative, noninvasive, and high‐resolution assessment of myocardial function and tissue integrity (Bulluck et al., 2018; Choi et al., 2001; Gerber et al., 2012; Kwong & Korlakunta, 2008; Lloyd & Gupta, 2005; Perazzolo Marra et al., 2011). Nevertheless, direct quantification of myocardial viability remains challenging. Global volumetric and functional parameters cannot fully capture this pathological remodeling as they are unable to characterize tissue properties (Haberkorn et al., 2017; Lang et al., 2021). Myocardial damage is routinely assessed in the clinic using late gadolinium‐enhanced MRI (LGE MRI). The physical properties of gadolinium (Gd) with shortening of long longitudinal relaxation times (T_1_), quick blood pool clearance times, and fast water exchange kinetics yield excellent imaging contrast and are generally considered safe (Boros et al., 2015; Flachskampf et al., 2011). Consequently, LGE MRI serves as one of the most prominent diagnostic and even prognostic markers of infarct size, but it cannot sensitively probe myocardial viability due to its lack of specificity (Jasmin et al., 2021; Skårdal et al., 2013). A number of pathological changes in tissue properties, including increases in extracellular space, interstitial edema, cell membrane rupture, or microvascular perfusion deficits can account for the changes in image contrast, with the time delay between onset of ischemia and Gd administration dictating the interplay between the aforementioned factors (Geelen et al., 2012; Hausenloy et al., 2010).

In recent years, the re‐emergence of manganese‐enhanced MRI (MEMRI) gained prominence for its heightened sensitivity and image contrast in cardiac tissue imaging. Manganese (Mn^2+^) enters viable cells through voltage‐gated calcium channels and can therefore be used to differentiate viable from nonviable myocardial cells. As an intracellular probe, Mn^2+^ allows for detailed quantification of myocardial function, providing insights into the dynamic alterations during ischemia and reperfusion in both animal and human studies. In previous MEMRI studies, investigations in animals have explored the correlations between Mn^2+^ uptake and several parameters, including (i) changes in cardiac inotropy, (ii) cardiac function alongside validated histological outcomes in ischemic models, and (iii) disruptions in calcium homeostasis, among others (Hu et al., 2001, 2004, 2005; Waghorn et al., 2008, 2011). Particularly in research on ischemic injuries, somewhat conflicting findings have emerged regarding enhanced signal intensity, even within the infarcted area, across different species. These inconsistencies include instances of heightened and transiently sustained signal intensity within the infarcted zone (Delattre et al., 2010; Hu et al., 2005; Saeed et al., 2000), absence of signal intensity elevation (Waghorn et al., 2011) or signal intensity increase followed by a rapid decay (Wen et al., 2019). These disparities underscore the critical necessity for further investigation into contrast enhancement mechanisms of Mn^2+^ and its link to myocardial viability (Singh, Joshi, Kershaw, et al., 2023; Spath et al., 2019, 2020, 2021; Wendland, 2004). Accurate assessment of myocardial viability is namely essential for guiding therapeutic strategies aimed at cardioprotection (e.g., early ischemic post‐conditioning) to limit adverse remodeling. Despite advances in diagnostic imaging, the ability to noninvasively evaluate tissue viability remains a significant clinical challenge.

This study primarily evaluates the potential of MEMRI for early detection of myocardial ischemic burden by assessing perfusion during ischemia and the initial phase of reperfusion. We employed a novel “in‐scanner” remote occlusion technique to induce ischemia during MRI scanning and utilized MEMRI to derive biomarkers for the early assessment of myocardial damage (Algoet et al., 2024). Additionally, we evaluated the ability of these biomarkers to predict the severity of functional decline following ischemic injury and its correlation with the extent of the resultant scar tissue. We hypothesized that MEMRI's specificity could provide a more accurate assessment of ischemic burden than LGE, offering a valuable tool for early prognosis and therapeutic decision‐making in myocardial infarction. Our findings could aid translating MEMRI into preclinical and clinical research, where it could guide early therapeutic strategies and improve patient outcomes following myocardial ischemic injury. By integrating noninvasive imaging with an innovative mouse model, this work seeks to expand the translational potential of MEMRI in preclinical research, offering a platform for “real‐time” functional and viability assessments that could inform novel, early therapeutic strategies.

## 2 METHODS

### 2.1 Experimental model of myocardial infarction

Mice (C57BL/6, male, *n* = 16, 10–12 weeks old) were included in this study. Naïve mice (*n* = 3) did not receive any surgery. The remaining mice (*n* = 13) underwent microsurgery to place a suture around the left anterior descending artery (LAD) which allowed remote occlusion using a novel “in‐scanner” setup (see Figure S1) as described previously (Algoet et al., 2024). In short, after induction of anesthesia using 2.5% isoflurane in pure oxygen (1 L/min flow rate) the animal was intubated and positioned supine on the base plate of a purpose‐built tool for remote occlusion/reperfusion within the MRI scanner. Following thoracotomy, the left anterior descending (LAD) coronary artery was identified, and a suture was placed around it before closing the chest. After completing the surgical procedure, an intraperitoneal catheter was secured in the animal's lower right quadrant to enable later contrast agent administration (see Contrast Agents for details). Prior to inducing ischemia, the animals were positioned within the center of the MR scanner, and standard cardiac views were established using scout MRI scans, and initial native images were acquired. Ischemia–reperfusion injury (IRI) was triggered remotely while the animal was positioned inside the MRI scanner and after the initial native images have been acquired (*n* = 7, *t* = 30 min). Animals have not been repositioned at any time after the initial preparation prior to the start of the MRI scan. Sham‐operated animals (assigned randomly after the initial native image acquisition, *n* = 6) received the same surgery with suture placement but without triggering ischemia. During surgery, ischemia/reperfusion, MR imaging, and recovery, the animal was kept warm, and the heart rate, respiration rate, and body temperature were closely monitored and maintained at physiological levels (450–600 min^−1^, 60–90 min^−1^, and 36–37°C, respectively) by regulating the isoflurane concentration and temperature. Ischemia was induced by inflating the vascular balloon catheter using a vascular balloon pump. Electrocardiogram (ECG) trace indicating elevation in the ST segment (part of the ECG waveform) was used as a marker of successful ischemia onset and was observed in all animals together with immediate increases in heart and respiration rates (ischemic animals). After 30 min of ischemia, the vascular balloon pump was unlocked and thus allowed subsequent LAD reperfusion. At the completion of the MRI, the animals were transferred to a bench where the tool was disassembled, and the animals were allowed to recover. The total time under isoflurane anesthesia did not exceed 2.5 h per time point. Male C57BL/6 mice were used to align with standardized ischemia–reperfusion models, minimizing hormonal variability (e.g., estrous cycle) that could confound acute outcomes. Focused on validating MEMRI and a novel surgical technique, this design prioritized controlled variables for direct comparison to prior studies.

Animal experiments were approved by the Ethical Committee for Animal Experimentation (ECD) at Katholieke Universiteit Leuven. All experiments were performed according to the guidelines and regulations set forth by the European Union concerning the welfare of laboratory animals as declared in Directive 2010/63/EU and its implementation into Belgian law (Royal Decree of May 2013) and a Decree by the Flemish Government (February 2017).

### 2.2 Contrast agents

Manganese chloride (MnCl_2_) (Sigma Aldrich, St. Louis, USA) was used as a probe to monitor dynamic alterations in tissue uptake after ischemia onset (day 0). Dosing used (0.1 mmol/kg) was based on a previous, in‐depth study (Jasmin et al., 2021). MnCl_2_ was diluted in saline (B. Braun Medical, Diegem, Belgium). The total volume of administered solution was less than 100 μL. An intraperitoneal catheter was secured in the lower right quadrant of the animal after surgical preparation. After initial image acquisition (native tissue contrast) and immediately after the start of ischemia, MnCl_2_ was delivered via this catheter. To measure the infarct size via LGE MRI 24 h after ischemia, gadolinium contrast (Gd‐DOTA) (Dotarem, Guerbet, France, batch: 17GS767B) was administered intraperitoneally (0.5 mmol/kg, i.p.) 20 min prior to the 24‐h follow‐up scanning.

### 2.3 Cardiac magnetic resonance imaging

Ischemic and sham mice underwent MRI on a 7T Biospec 70/30 MR scanner (Bruker PCI, Ettlingen, Germany) at four time points (baseline, day 0, 1, and 28). Naïve animals were only scanned at baseline and after MnCl_2_ injection (day 0 time point). An 86 mm quadrature transmit coil combined with a rat brain surface receive‐only coil (T20010V3, Bruker PCI) placed over the chest of the animals was used with the animals positioned supine. Inhalation anesthesia was maintained at 1.5% isoflurane in 1.5 L/min O_2_ throughout all MRI experiments. Body temperature, respiration, and cardiac rate were maintained at 36–37°C, 60–90 min,^−1^ and 450–600 min^−1^, respectively. Core temperature was maintained with warm air being blown through the magnet bore to the surroundings of the animal (day 0) or warm water in a closed circuit within the animal bed (all other time points). ECG signal was derived from three needle electrodes placed in the fore and hind paws. Respiratory monitoring was performed with a balloon pressure transducer positioned underneath the animal's abdomen. Temperature monitoring was performed with a rectal temperature probe. Cardio‐respiratory‐temperature monitoring was performed using an MR‐compatible system (SA Instruments, NY, USA).

#### 2.3.1 MEMRI and T_1_ mapping (day 0, immediately after surgery)

A 3D retrospectively gated T_1_‐weighted FLASH sequence (IntraGate, ParaVision 6.0.1, Bruker PCI, Ettlingen, Germany) in short‐axis orientation was used at day 0 (prior to MnCl_2_) for tissue characterization via T_1_ mapping. A variable flip angle (VFA) approach was employed, where the flip angles (FA) used were [2, 5, 8, 11, 14] degrees, and the echo time (TE) and repetition time (TR) were 2.3 ms and 11.1 ms, respectively. Imaging was performed with a matrix size of 120 × 110 × 10 and a field of view (FOV) of 28 × 25 × 8 mm^3^. Following the native T_1_ map acquisition, ischemia was triggered remotely without repositioning the animal nor the geometry and was followed by MnCl_2_ administration. Immediately after, the data acquisition continued by utilizing the same sequence T_1_‐weighted FLASH sequence at FA = 14° to track the contrast uptake while maintaining the set image contrast. Data were continuously acquired for 66 min (11 sequence repetitions), which included the initial 30 min of LAD occlusion followed by 36 min of reperfusion.

#### 2.3.2 Cine imaging (baseline and 28 days post‐surgery)

Retrospectively gated 3D T_1_‐weighted sequence (described above) was used with an increased oversampling factor (factor = 200) allowing to reconstruct the data into 20 cardiac frames for volumetric analysis. Additionally, a 2D black‐blood sequence (3 slices: apex, mid‐ventricle, base) with parameters matching LGE parameters (except navigator FA = 90° for blood nulling) enabled wall thickness measurements.

#### 2.3.3 LGE MRI (24 h post‐surgery)

LGE imaging was performed using a 2D retrospectively gated T_1_‐weighted FLASH (IntraGate, Bruker PCI) in axial orientation. Parameters included: TE/TR = 2.3/11.1 ms, flip angle = 45°, matrix = 120 × 110, FOV = 28 × 25 mm^2^, 7–8 contiguous slices (1 mm thickness), and oversampling = 200. Images were acquired ~20 min post‐contrast injection, retrospectively reconstructed into 20 cardiac frames.

### 2.4 Image analysis

Image preprocessing, manual contouring of the LV, and processing of LGE and MEMRI data was performed using custom scripts in MATLAB (MathWorks, Natick, USA). LGE‐derived area at risk (AAR), expressed as a percentage of the left ventricle (%LV), was defined as the area exceeding a conventional threshold set to the mean signal intensity + 2 standard deviations (SD) derived from a reference septal region of interest (ROI). Perfusion deficit analysis via MEMRI data, expressed as %LV, was conducted using two distinct approaches: (i) Threshold‐based method: Direct comparison with LGE‐derived AAR, applying the same thresholding technique (septal mean + 2 SD) to ischemia end time point data (30 min post‐occlusion). (ii) Linear slope method: Pixel‐wise calculation of signal intensity slopes during ischemia, obtained by dividing the difference between final and initial native signal intensities by the ischemic time interval. Perfusion deficit area was defined as the percentage of pixels with a nonpositive slope coefficient. Analysis of cardiac function was performed using Segment (Medviso, Lund, Sweden) (Heiberg et al., 2010). Manual endocardial and epicardial segmentation of the left ventricle (LV) at end‐diastolic and end‐systolic cardiac phases yielded end‐diastolic volume (EDV) and end‐systolic volume (ESV), from which ejection fraction (EF) was calculated. Wall thickness was quantified using analogous manual delineations of the mid‐ventricular slice in black‐blood MRI sequences. For T_1_ relaxation parameter analysis, custom MATLAB scripts were employed, incorporating available code for T_1_ mapping and bias field correction (Chunming, 2025; Ramos‐Llordén et al., 2018).

### 2.5 Histology

Sirius red (SR) staining was performed 28 days after surgery to outline the extent of scar tissue formation. Bright red areas mark the necrotic core of the infarct by collagen deposition, whereas the orange parts mark the viable tissue. Briefly, following the completion of the last MRI scan, mice were sacrificed, the hearts rapidly harvested, and fixed for further processing in Zinc Formalin Fixative (cat N° Z2902, Sigma‐Aldrich, St. Louis, USA). Sirius red staining was performed as previously described (Rittié, 2017). The whole heart was longitudinally divided into four sections and then cut perpendicularly in short axis orientation (see Figure S2). One representative tissue slice from each section was used to measure the infarct area, expressed as a percentage of the left ventricle (%LV), using a thresholding macro in ImageJ software (Kammerer et al., 2024; Schneider et al., 2012).

### 2.6 Blood sampling

Cardiac injury was also assessed using high‐sensitivity Troponin I ELISA. Blood samples were collected at 24 h after surgery for IRI (*n* = 7) and sham (*n* = 6) animals, prior to the LGE scan, and Hs‐TnI levels were determined on plasma samples by ELISA (CTNI‐1‐HSP, Life Diagnostics, Stoke‐on‐Trent, United Kingdom).

### 2.7 Statistical analysis

All statistical analyses were performed using GraphPad Prism (v10.4.0). All data is presented as mean ± standard error of mean (SEM) if not indicated otherwise. One ischemic animal was excluded from the analysis of MnCl_2_ uptake due to technical error. One ischemic and two sham animals were excluded from T_1_ analysis due to technical error. All experimental data were subject to Shapiro–Wilk test to assess normality and inform tests, as appropriate. Differences in ROI MnCl_2_ uptake were compared using 2‐way ANOVA. Comparative assessments of ischemic burden between groups were tested using 2‐tailed *t*‐test with Welch's correction. Friedman test with Dunn's post hoc (IRI group) or ANOVA followed by Tukey's post hoc test (sham group) was used for a direct matched comparison across metrics. Spearman correlation was used to check agreement between early metrics and long term remodeling. For characterization of the animal model, 2‐way ANOVA followed by Tukey's post hoc test was used to test cardiac functional data between multiple groups and unpaired, 2‐tailed *t*‐test with Welch's correction was used to assess the difference in serum markers and tissue relaxation parameters between ischemic and sham animals. Significance for all panels: *p < 0.05; **p < 0.01; ***p < 0.001; ****p < 0.0001.

## 3 RESULTS

**FIGURE 1:**
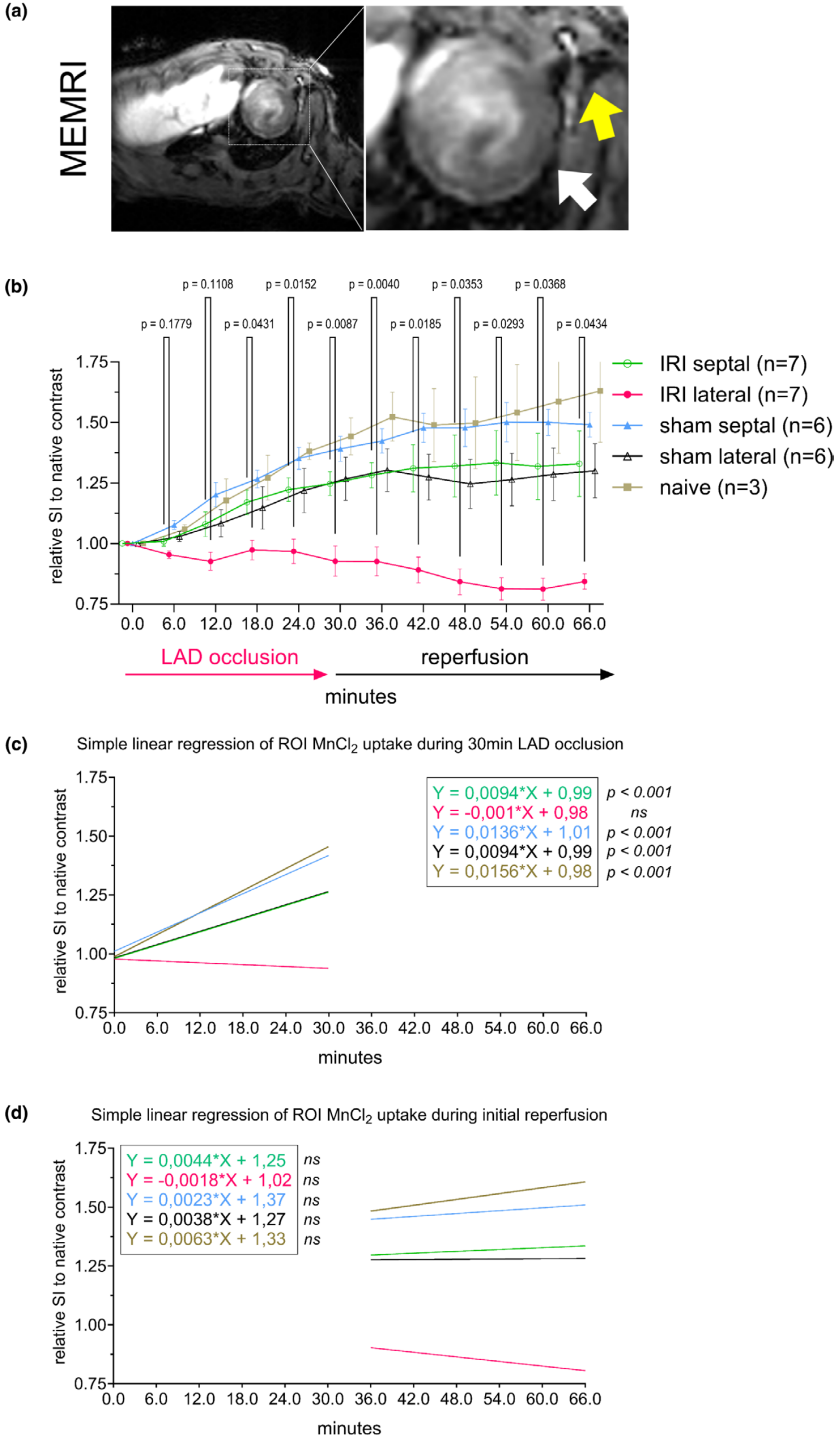
MEMRI during remotely induced ischemia outlines the hyperperfused area. (a) Short‐axis mid‐ventricular slice is shown (raw signal intensity; a.u.) at 30 min of LAD occlusion. White arrow points to the lack of increased signal intensity observed in the lateral region of the myocardium, depicted as the ischemic zone. Yellow arrow points to the suture placement. (b) ROI analysis revealed difference in contrast dynamics in the lateral regions compared to septal regions in ischemic animals. (c) Linear regression analysis of signal intensities between the start and end of ischemia. (d) Linear regression analysis of signal intensities between the start and the initial 30 min of reperfusion. ROI, region of interest; SI, signal intensity.

Figure 1 illustrates the utility of MnCl_2_ as a perfusion and potential viability tracer during ischemia and early reperfusion using a remote “in‐scanner” occlusion technique. T_1_‐weighted MRI (Figure 1a) revealed hypointense signal intensity in the ischemic zone (reduced Mn^2+^ uptake) and hyperintensity in the viable septal region (robust Mn^2+^ uptake). The contrast difference generated due to the LAD occlusion was observable after the first acquired data point (6 min post‐occlusion, data not shown). Temporal analysis (Figure 1b) showed distinct MnCl_2_ uptake dynamics in the lateral region of the ischemia–reperfusion injury (IRI) group compared to the septal region in the IRI group (*p* < 0.05). Minimal signal changes during initial reperfusion suggested that MEMRI dynamics are primarily observable during ischemia and the initial wash‐in period (0–30 min). Limited myocardial repositioning was observed at the inflation/ deflation stages of the balloon catheter (initiation of ischemia and reperfusion, respectively), prompting the separation of the acquired time dynamics into two separate sets: time of ischemia and time of initial reperfusion. During ischemia, simple linear regression analysis (see Figure 1c,d) revealed a significant non‐zero slope in all ROI except for the lateral ROI in ischemic animals (IRI lateral: ns). Specifically, significant slopes (*p* < 0.0001) were observed in the septal ROI of ischemic animals (IRI septal) and in both lateral and septal ROIs of sham and naïve animals. However, at the initial reperfusion, no significant differences in slope were detected across the ROIs and animal groups.

**FIGURE 2:**
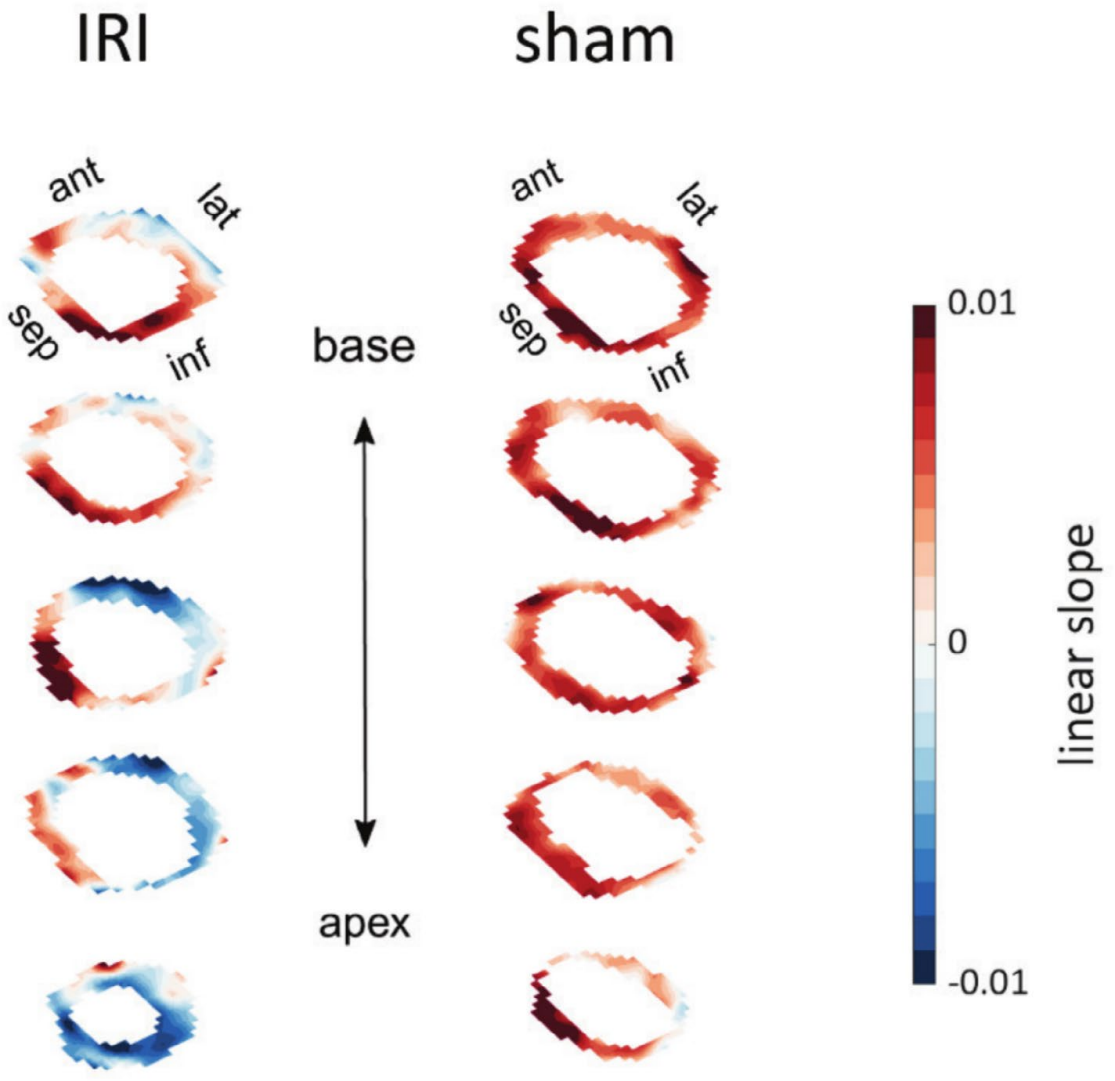
Volumetric mapping of ischemic perfusion deficit during LAD occlusion using MEMRI. Representative 3D perfusion maps of the delineated myocardium in an ischemic animal (left) and a sham animal (right), acquired during remote temporary LAD ligation. Perfusion deficit was quantified using the slope criterion (ΔSI = SI_*t*=30min_–SI_*t*=0min_). The volumetric overlay highlights the spatial extent of perfusion impairment in the ischemic animal, localized to the LAD territory. Data are displayed in short‐axis orientation (ant, anterior; inf, inferior; lat, lateral; sep, septal; SI, signal intensity).

**FIGURE 3:**
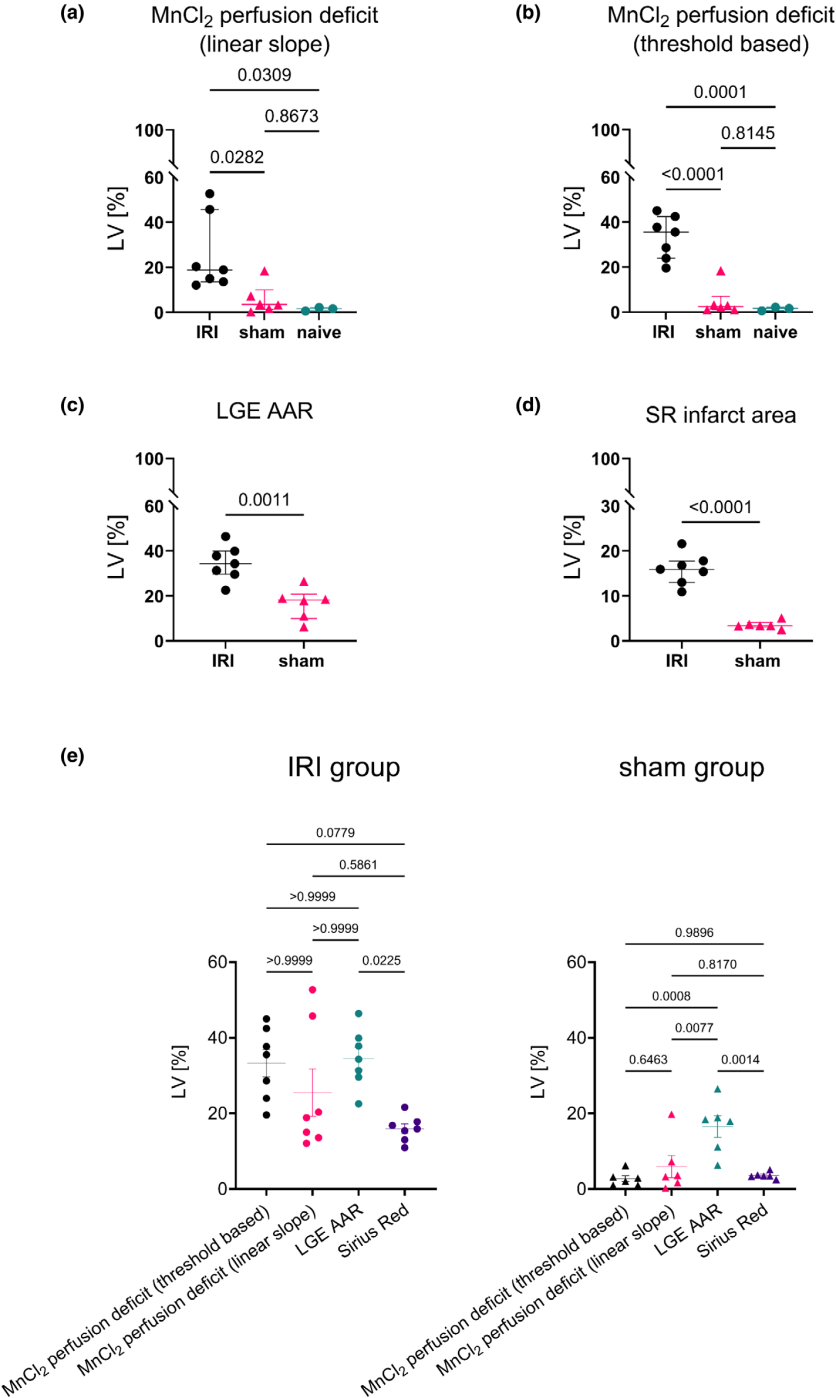
Comparative assessment of ischemic burden across imaging and histological metrics. (a) MEMRI‐derived perfusion deficit quantified via the linear slope metric demonstrated significant differences between ischemic animals and both sham and naïve groups, reflecting ischemia‐specific Mn^2+^ uptake impairment. (b) Similarly, threshold‐based MEMRI analysis distinguished ischemic animals from sham and naïve groups, corroborating the linear slope findings. (c) Late gadolinium enhancement (LGE)‐defined area at risk (AAR) confirmed elevated ischemic injury in the IRI group versus sham controls. (d) Histological assessment (Sirius red staining) validated significant myocardial damage in ischemic animals compared to sham. (e) In the IRI group LGE‐AAR overestimated infarct size relative to SR, while MnCl_2_‐derived perfusion deficits showed no significant discrepancy from LGE or SR. Sham animals exhibited significant overestimation of injury by LGE versus MnCl_2_ threshold‐based perfusion deficits and linear slope versus LGE, despite limited histological evidence of scar formation versus LGE.

Using a pixel‐wise linear slope analysis, the whole volumetric extent of ischemia could be delineated by comparing signal intensities just before and at 30 min of occlusion. Figure 2 demonstrates the extent of perfusion deficit observed in an ischemic animal (left) versus a sham animal (right). The delineated myocardium in short‐axis orientation highlights the difference in contrast uptake within the same slice as well as the long‐axis of the heart. A large positive slope, indicating high contrast uptake, is visible in the septal region in both animals, with a negative slope showing decreased signal intensities due to vessel occlusion seen in the area primarily perfused by LAD (lateral wall of the ischemic animal). Figure 3 further compared MnCl_2_‐derived perfusion deficits (linear slope and threshold‐based methods), LGE‐AAR, and histology. Analysis showed all metrics distinguished ischemic from sham animals (MnCl_2_‐linear slope: *p* = 0.0282; MnCl_2_‐threshold: *p* < 0.0001; LGE: *p* = 0.0011; SR: *p* < 0.0001). MnCl_2_ perfusion deficits also differentiated ischemic from naïve animals (*p* < 0.0001). Mean values of perfusion deficits were 33.25% (threshold based), 25.46% (linear slope), 34.54% for LGE AAR, and 15.91% for infarct size measured with SR.

**FIGURE 4:**
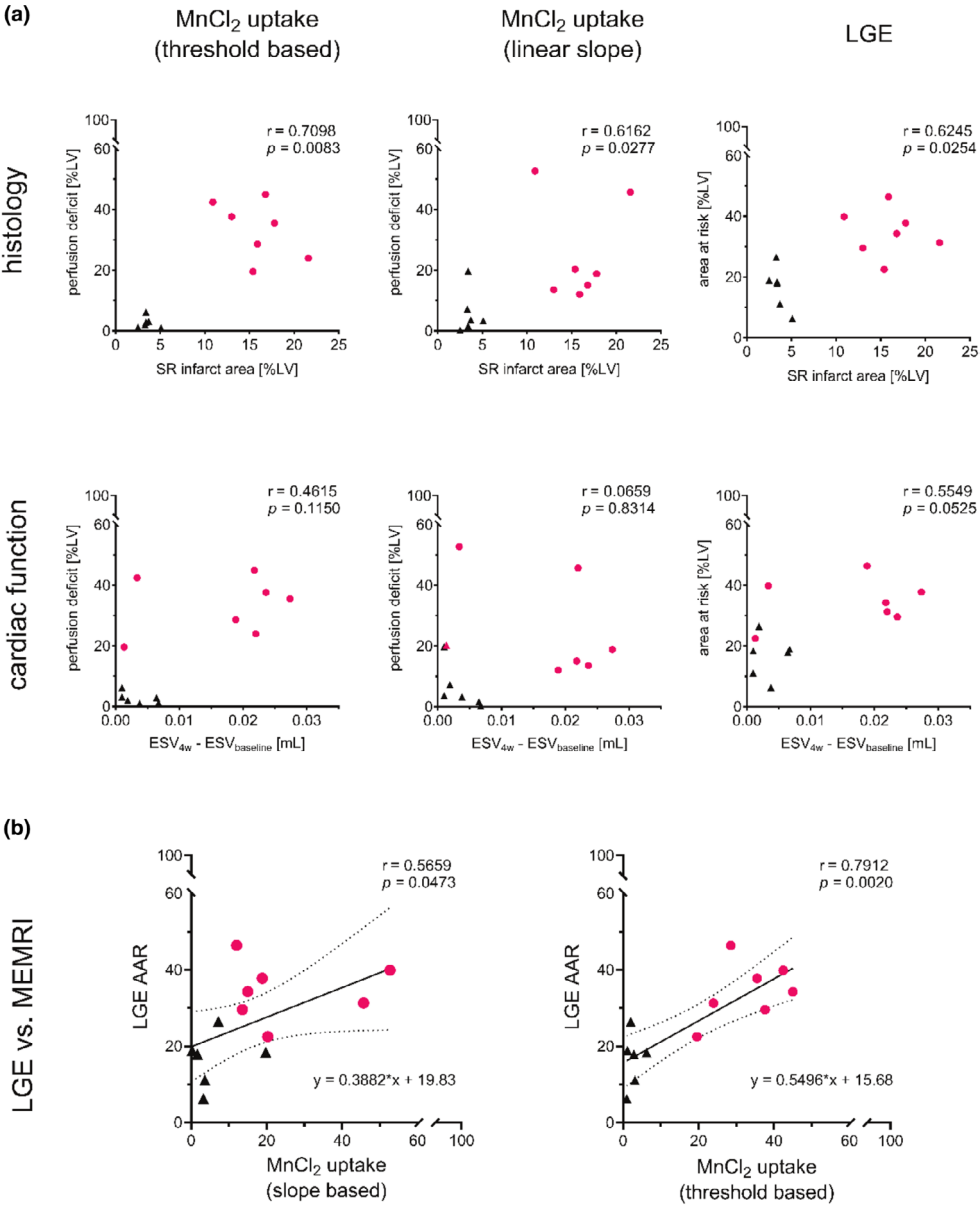
Correlation of early imaging metrics with histological and functional outcomes. (a) Early assessment of myocardial damage using MnCl_2_ perfusion deficit (linear slope and threshold‐based methods) and LGE‐derived area at risk was correlated with histologically quantified infarct size (SR) and long‐term cardiac dysfunction (ΔESV: End‐systolic volume at 4 weeks vs. baseline). (b) Correlation of myocardial damage between both imaging markers of acute myocardial injury (LGE vs. MEMRI) with simple linear regression line fit (dotted lines mark the 95% fitting confidence).

In matched comparisons (Figure 3e), LGE‐AAR overestimated infarct size relative to SR in both ischemic (*p* = 0.0225) and sham groups (*p* = 0.0014). MnCl_2_ perfusion deficits showed no discrepancy with LGE or SR in ischemic animals. However, LGE overestimated the extent of injury compared to perfusion‐derived metrics in sham controls (threshold: *p* = 0.0008; linear slope: *p* = 0.0077). Figure 4a indicates that MnCl_2_‐derived metrics, as well as LGE‐AAR, correlated strongly with final histologic infarct size. In contrast, the predictive power for adverse ventricular remodeling (ΔESV at 4 weeks vs. baseline) remained modest (SR vs. ΔESV, *r* = 0.8116, *p* = 0.0012). Correlation analysis between both imaging markers of acute myocardial injury (LGE vs. MEMRI) reveals a link between the two measures at different times post‐injury (threshold: *r* = 0.7912, *p* = 0.0020 and slope: *r* = 0.5659, *p* = 0.0473). Simple linear regression reports the regression line offset at 15.68 and 19.83 %LV for threshold and slope‐based MEMRI analysis, respectively.

**FIGURE 5:**
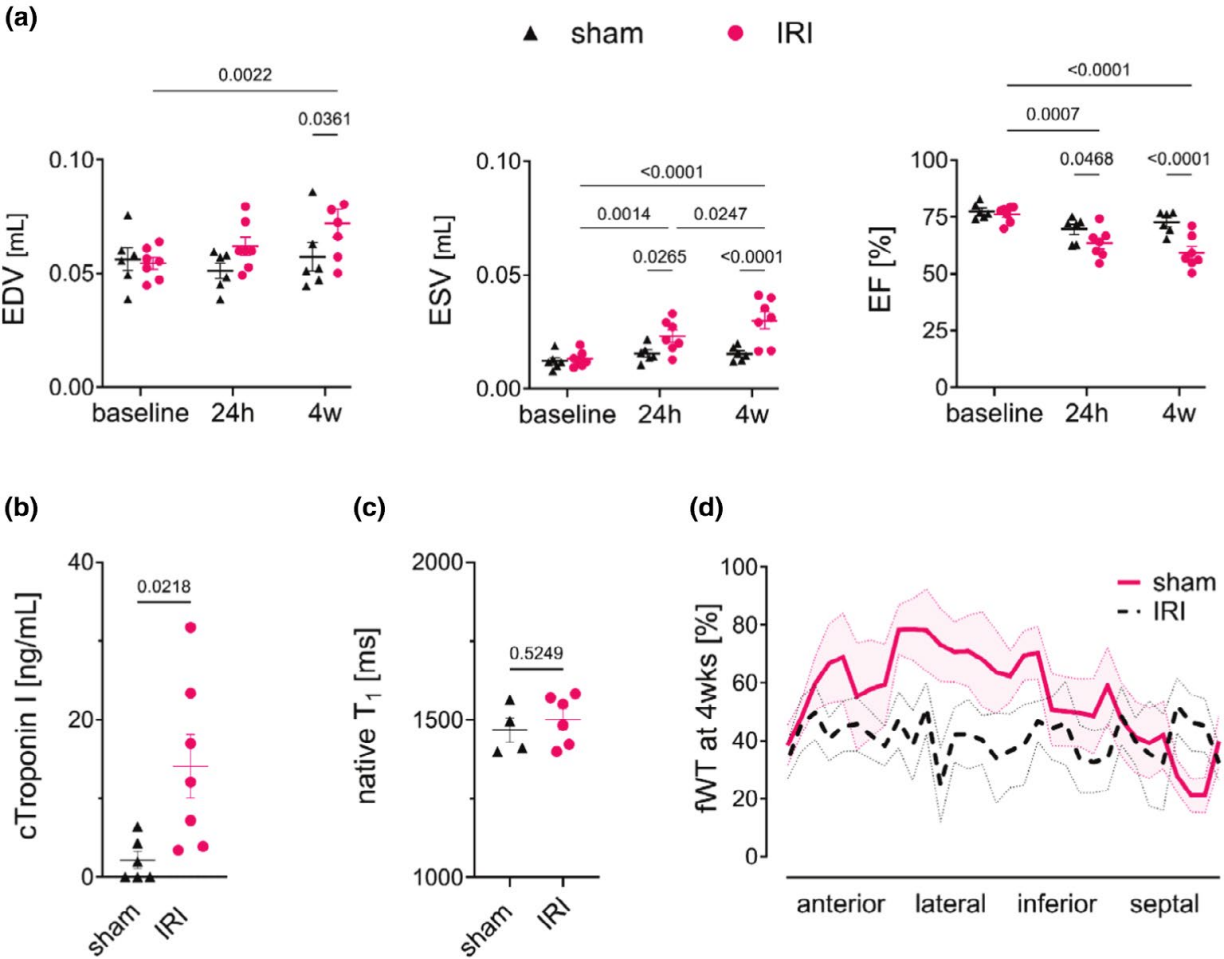
Longitudinal assessment of cardiac remodeling and functional outcomes. (a) Cardiac cine MRI revealed significant left ventricular (LV) remodeling 4 weeks after remote LAD occlusion. While baseline end‐systolic (ESV; *p* = 0.7768) and end‐diastolic (EDV; *p* = 0.8025) volumes did not differ between groups, ischemic animals exhibited marked systolic dysfunction at follow‐up, whereas sham‐operated animals maintained stable function. (b) Serum cardiac troponin I levels 24 h post‐surgery were significantly elevated in the IRI group versus sham. (c) Pre‐occlusion native T_1_ relaxation times showed no difference between IRI and sham groups. (d) Segmental fractional wall thickening (fWT) at 4 weeks demonstrated impaired contractility in the IRI group (red dotted line), particularly in the lateral wall (histologically confirmed infarct zone), versus sham (solid line). Data are presented as segmental averages with shaded 5% error bands, highlighting regional dysfunction.

Volumetric analysis (Figure 5a) confirmed maladaptive remodeling in the IRI group, with mean values of elevated EDV (69.6 ± 5.9 vs. 58.3 ± 7.5 μL, *p* = < 0.05) and ESV (27.8 ± 3.9 vs. 15.9 ± 1.3 μL, *p* = < 0.0001) and reduced EF (61.37 ± 3.16% vs. 71.92 ± 2.22%, *p* = <0.0001) compared to sham at 28 days. Serum troponin levels 24 h post‐IRI (13.1 ± 3.6 ng/mL, *p* < 0.05 vs. sham; Figure 5b) confirmed acute injury. Native T_1_ relaxation times acquired after preparation of the animals and before LAD occlusion did not differ between IRI and sham groups (*p* = 0.5249, Figure 5c). At 4 weeks, fractional wall thickening (fWT) was visually impaired in the IRI group, particularly in the lateral wall (LAD territory; Figure 5d).

## 4 DISCUSSION

In this study, we present the first application of a remote LAD occlusion‐reperfusion model performed inside an MRI scanner combined with manganese‐enhanced MRI to dynamically track acute ischemic burden and perfusion changes in mice in vivo. This approach enabled real‐time visualization of dynamic perfusion deficits during ischemia and reperfusion, while simultaneously quantifying the immediate ischemic burden using two complementary methods: the kinetic slope of Mn^2+^ uptake and threshold‐based analysis. Notably, MnCl_2_ perfusion derived threshold‐based method provided a direct comparison to LGE, the current clinical gold standard for infarct size estimation, while circumventing LGE's limitations in overestimating infarct size in non‐ischemic contexts. We established correlations between Mn^2+^ uptake patterns during acute ischemia and subsequent histologic infarct size, as well as ventricular remodeling and functional decline at 4‐week follow‐up. These findings position MEMRI not only as a sensitive diagnostic tool for acute ischemic injury but also as a potential prognostic biomarker for post‐infarct remodeling. The strong concordance between MEMRI‐derived metrics and endpoint histology underscores its preclinical potential for evaluating and guiding early therapeutic decisions, particularly in assessing myocardial viability during the critical window following ischemia–reperfusion injury. Early, accurate viability assessment is pivotal for optimizing interventions aimed at salvaging ischemic myocardium, such as reperfusion therapies or cardioprotective agents. While our results highlight MEMRI's advantages over LGE in specificity and dynamic tracking, further studies are needed to validate these findings in larger cohorts and diverse ischemic models. Taken together, our work confirms MEMRI as a versatile platform for bridging preclinical mechanistic insights with clinical needs in ischemia management.

Our results provide a comprehensive comparison of MnCl_2_‐derived perfusion deficits, LGE‐AAR, and SR histology for assessing myocardial ischemia and infarction. The results demonstrated that both linear slope and threshold‐based MnCl_2_ perfusion metrics effectively distinguished ischemic from sham‐operated animals. Notably, while LGE‐AAR and SR infarct size also robustly discriminated ischemic injury, direct comparisons revealed an overestimation of infarct size by LGE‐AAR relative to SR in both ischemic and sham groups. This overestimation is consistent with prior findings indicating that LGE‐AAR reflects the myocardial territory at risk, which may include both infarcted and salvageable myocardium as described above, and its extent will vary depending on the time of investigation, whereas SR provides a definitive histological delineation of scar tissue (Nikolaou et al., 2024; Skårdal et al., 2013). MEMRI‐derived perfusion deficit metrics showed a similar distinction between groups and were comparable to the LGE‐based assessment in the ischemic animals. However, consistent with previous observations, LGE reported a higher area at risk, as evident in sham animals, highlighting the lack of specificity of this contrast agent (see Figure S3) (Geelen et al., 2012).

Through quantification of Mn^2+^ uptake during ischemia, we are not only able to delineate the ischemic zone, but also show predictive potential for the final infarct size and the degree of failure in cardiac function, similar to Hu et al. (2004). Important to note, our investigational time window was focused on the immediate post reperfusion phase in the IRI model instead of the delayed subacute phase (1 week) stage in a permanent ligation model. These findings contribute to the growing preclinical evidence supporting the high sensitivity of Mn^2+^ uptake for identifying ischemic zone borders at an early stage. Correlation analyses revealed moderate associations between MnCl_2_‐derived metrics, LGE‐AAR, and final histologic infarct size, supporting their utility in characterizing myocardial injury. Nevertheless, the predictive power of these imaging approaches for adverse ventricular remodeling, as assessed by changes in end‐systolic volume (ΔESV) at 4 weeks versus baseline, remained modest, possibly due to smaller infarct sizes observed in our animal model. This suggests that while imaging biomarkers provide crucial structural insights, additional functional or molecular markers may be required to improve prognostic accuracy. Overall, our data highlight the complementary nature of each modality as they interrogate distinct aspects of the injury process at different investigation time points: Mn^2+^ MR imaging excels in early detection of perfusion deficits and viable myocardium, LGE MRI characterizes subacute extracellular changes and evolving scar tissue, while SR histology confirms chronic fibrotic remodeling. This integrative approach underscores the value of combining phase‐specific imaging modalities to achieve a comprehensive understanding of myocardial injury evolution and is consistent with previously reported findings from both preclinical and clinical studies (Spath et al., 2020; Spath et al., 2021; Wendland, 2004). This early depiction of the ischemic zone is important for tracking the changes during a longitudinal assessment of myocardial damage as only the border region between the ischemic and non‐ischemic tissues has the potential to recover. As the focus of these experiments was on the validation of the animal model, no intermediate follow‐up time points were included to investigate this.

A major advantage of our experimental approach, combining remote LAD occlusion with MEMRI, is its ability to characterize myocardial tissue immediately before and during ischemia, as well as throughout reperfusion, to distinguish the confounding effects from surgical trauma. The benefit of determining whether the LAD is to be occluded and while the animal is already positioned in the MR scanner allows us to obtain, for example, exact tissue texture properties after thoracotomy but prior to inducing ischemia. Our results confirmed that surgical trauma itself does not correspond to myocardial damage observed due to ischemia reperfusion injury. The average native T_1_ value across both groups (IRI and sham, 1490 vs. 1487 ms, respectively) is in agreement with values reported for healthy animals in similar studies (Coolen et al., 2011; van den Boomen et al., 2019). Our result aligned with findings of Waghorn et al. (2008) on the observation that thoracotomy has no impact on the absorption of Mn^2+^ as we evidenced the undisrupted MnCl_2_ uptake pattern in the lateral regions of sham animals.

In a preceding study by Hu et al., the applicability of Mn^2+^ for examining instantaneous cellular calcium regulation was reported within a canine model of IRI in “real time” with MRI (Hu et al., 2005). Our murine model findings corroborate prior observations on the temporal dynamics of Mn^2+^ uptake, thereby validating the utility of MEMRI for near‐real‐time monitoring of myocardial ischemia. Spatial heterogeneity in Mn^2+^ uptake (lateral vs. septal myocardium) enabled real‐time spatial delineation of the ischemic zone during coronary occlusion. In our study, however, MEMRI data acquired during ischemia reflects indications of perfusion deficits rather than cellular viability, as manganese contrast was administered post‐ligation of the left anterior descending artery. Additionally, contrast dynamics were primarily observed within the first 30 min post‐administration, with minimal changes thereafter, limiting the assessment of tissue viability beyond this time point. These insights on time dynamics will inform the design of future studies to optimize imaging protocols for concurrent evaluation of perfusion and viability during ischemia–reperfusion transitions.

We have characterized the previously developed remote IRI model using cMRI. Combined, results of cardiac functional MRI and histology confirmed that the failing cardiac function can exclusively be attributed to myocardial ischemia generated by LAD occlusion inside the MR scanner, as the total surgery time is the same in both groups (IRI and sham animals). Cardiac functional parameters obtained at 28 days post‐surgery, together with measurements of scar tissue formed are comparable to other studies using similar animal models (Delattre et al., 2010; Eckle et al., 2006; van den Boomen et al., 2019). This degradation in cardiac function stipulated that remote occlusion of the LAD is suitable as a robust experimental model to study ischemia reperfusion injury in vivo in mice also on a longitudinal scale. In addition, indications of contractility compensation appear to be arising in the septal part, as distinguished by wall thickness measurements. However, this would need to be further characterized on a longitudinal basis with measurements at later time points. Lack of collagen deposition observed in the sham group confirmed by histology, together with no detected differences in native tissue characterization also demonstrated, that cardiac surgery alone (i.e., thoracotomy with suture placement) does not alter myocardial tissue viability nor function. Mechanical interventions designed to mitigate ischemic burden following ischemia, specifically ischemic post‐conditioning, will be further investigated using this experimental setup. This will involve the synchronized acquisition of cMRI data to comprehensively assess tissue function and composition.

### 4.1 Clinical relevance

Clinical studies have shown that MEMRI effectively assesses myocardial viability and function, particularly by identifying the peri‐infarcted region (a key area for potential recovery), while also exhibiting high reproducibility potential in investigations of myocardial calcium handling (Singh, Joshi, Kershaw, et al., 2023; Spath et al., 2021; Wen et al., 2019; Wendland, 2004). Our preclinical results support MEMRI's potential in cardiac research; however, further studies investigating the paradigm of myocardial contrast enhancement and its link to myocyte viability are needed.

Currently, no clinical grade Mn^2+^ based contrast agent is widely available, even though approval for human use was granted (Spath et al., 2019). Here, the choice was made to use MnCl_2_, due to its low cost, ease of distribution and through reports on its biocompatibility upon administration in small animals. Previously published reports on Mn^2+^ cardiotoxic effects describe within minutes fading cardiac depression effects with generally no reports of cardiac toxicity. However, long‐term safety data is currently still lacking (Jynge et al., 2020; Lamonzie et al., 2022; Singh et al., 2023; Singh, Joshi, Kershaw, et al., 2023). Although we did not perform any additional physiological measurements, Jasmin et al. (2021) report through in vitro and in vivo findings on a variety of Mn^2+^ concentrations observing limited transient cardiac suppressing consequences. Upon administering the lowest dosage evaluated in that study (0.1 mM i.p.) we confirm observing reduced ECG signal amplitude, with temporary increases in heart and respiration rates, however, those effects faded within 30 s after slow administration of MnCl_2_ (data not shown). Additionally, as we performed a longitudinal follow up of 28 days, no (patho‐) physiological side effects were observed in the sham nor IRI group (data not shown). Nevertheless, it might be beneficial to lower the dosage even further and search for the threshold of minimal dosage necessary while still reaping the contrast agents benefits.

### 4.2 Limitations and outlook

This study has some limitations. First, the longitudinal follow‐up of all animals prevented histological comparisons of LGE and Mn^2+^ infarct delineations at earlier time points. Second, Mn^2+^ was administered immediately after occlusion onset, similarly as in a previous study in canine (Hu et al., 2005), to monitor contrast uptake dynamics during ischemia and reperfusion. This administration protocol lead to interpreting the acquired data during the LAD occlusion as perfusion based. However, as the circulation of the contrast agent was observed to be drastically reduced after the initial 30 min post administration (coinciding with the end of the ischemic period), we were not able to investigate contrast enhancement mechanisms and their link to calcium handling during the initial reperfusion period (30 min + post administration). To address these limitations, future studies should systematically evaluate alternative Mn^2+^ administration timings, such as initiating administration at the onset of reperfusion. This strategy could leverage initial high wash‐in dynamics to characterize the ischemic zone and assess myocardial Mn^2+^ uptake dynamics immediately post‐reperfusion. Furthermore, monitoring Mn^2+^ blood concentrations over time would enable direct quantification of myocardial uptake.

## 5 CONCLUSIONS

The growing body of preclinical as well as clinical research utilizing Mn^2+^ in cardiac MRI applications demonstrates the wide applicability and potential of this intracellular contrast agent. We show that in mice, a species widely used in preclinical research and easily amenable to genetic manipulation, administration of low doses of Mn^2+^ enables visualization and robust quantification of the ischemic zone. Our quantitative data on Mn^2+^ uptake patterns in the myocardium during ischemia add preclinical evidence that MEMRI serves as a compelling biomarker for evaluating myocardial status during the acute ischemic burden and for enabling projection of myocardial functional and structural remodeling at follow up.

## AUTHOR CONTRIBUTIONS

M.P., M.A., W. G., S. J., W. O., and U. H. conceived and designed research, interpreted results of experiments, edited and revised manuscript, and approved final version of manuscript. M.P. and M.A. performed experiments. M.P. analyzed data, prepared figures, and drafted manuscript.

## FUNDING INFORMATION

The research was supported by research grants of KU Leuven (C14/20/095) and the Research Foundation‐Flanders (FWO G0A7722N). M. Algoet was supported by the Research Foundation Flanders Fellowship Grant (11A2423N).

## CONFLICT OF INTEREST STATEMENT

Authors declare no conflict of interest.

## Supporting information


Figures S1–S3.

## Data Availability

The datasets used and analyzed during the current study are available upon a reasonable request.
