# Supplementary material for: Manganese‐enhanced MRI during remotely induced myocardial ischemia reperfusion injury in male mice
Source: Physiol Rep. 2025 Jul 3;13(13):e70442. doi: 10.14814/phy2.70442 (PMC12223404; doi:10.14814/phy2.70442)
Supplement: Supplementary file 1 — Figures S1–S3. [file PHY2-13-e70442-s001.docx]

**SUPPLEMENTAL MATERIAL**


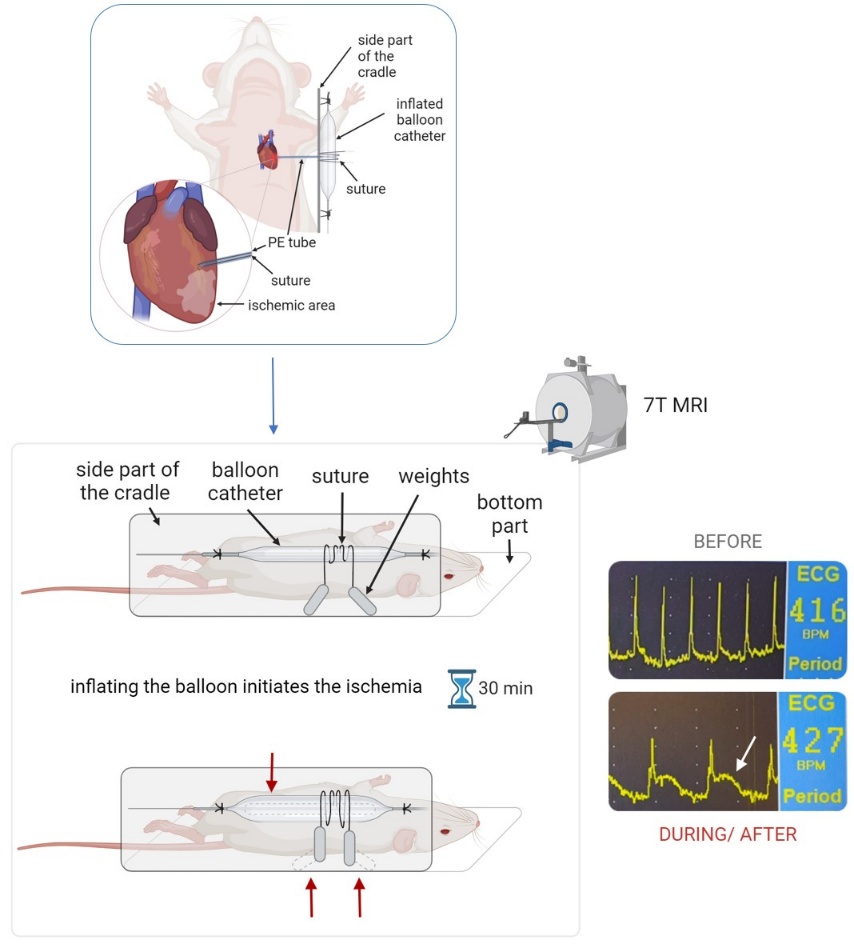


Supplementary Figure 1: Schematic illustration of the tool for remote LAD occlusion inside the MR scanner. ECG trace (right side) just before and during remote LAD occlusion inside the MR scanner shows the typical elevation in the ST segment (pointed arrow). Created with BioRender.com.


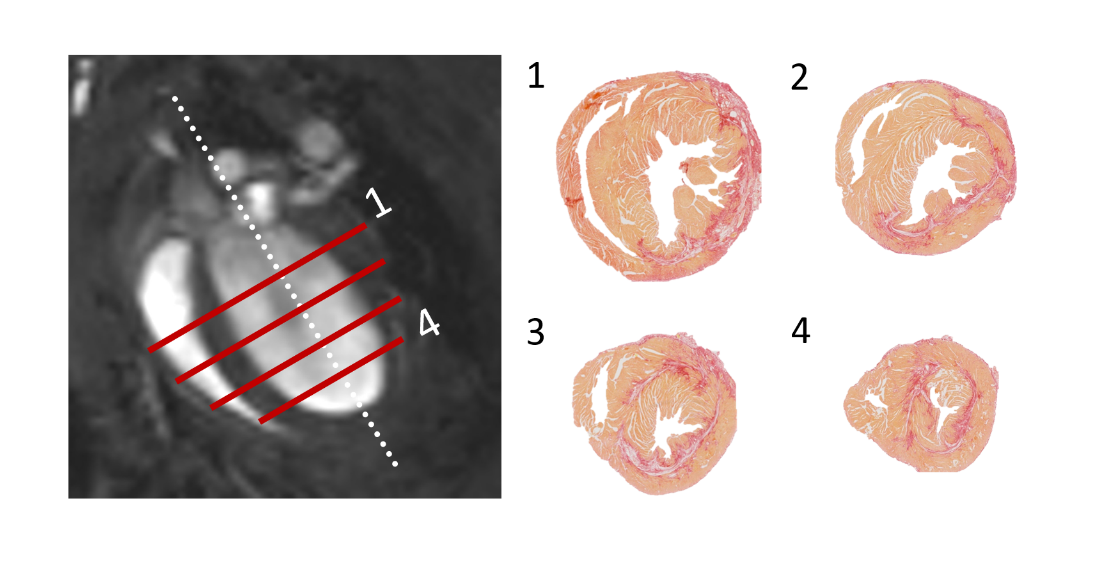


Supplementary Figure 2: Illustrative example of Sirius red staining with slices from each of the four sections (right, spacing of ~1 mm between sections) of an ischemic animal with the slice positioning with respect to the long axis of the myocardium (left, four chamber scout MRI).


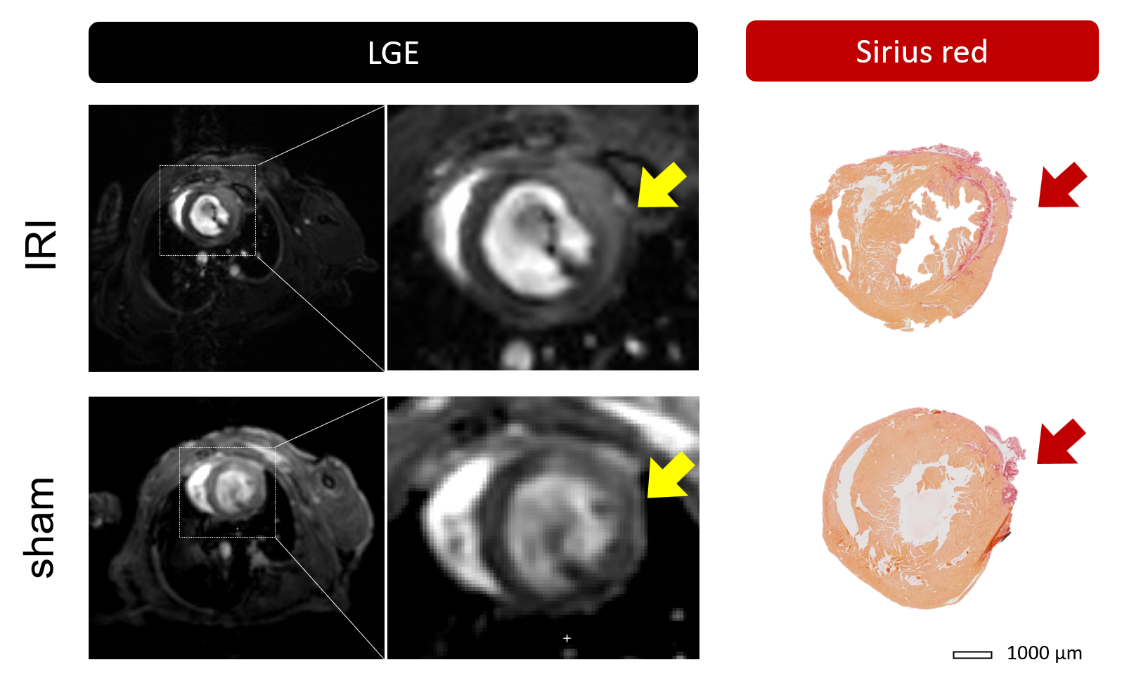


Supplementary Figure 3: LGE MRI (at 24 hour follow-up) outlines the AAR (top row: ischemic animal, bottom row: sham animal). Yellow arrows (both rows) point to the area of increased signal intensity observed in the lateral region of the myocardium. Sirius red outlines the extent of the scar tissue formed (red arrows, both rows). Raw LGE signal intensities are displayed with individual arbitrary contrast setting for illustration purposes.
